# HDAC Inhibition Induces Increased Choline Uptake and Elevated Phosphocholine Levels in MCF7 Breast Cancer Cells

**DOI:** 10.1371/journal.pone.0062610

**Published:** 2013-04-23

**Authors:** Christopher S. Ward, Pia Eriksson, Jose L. Izquierdo-Garcia, Alissa H. Brandes, Sabrina M. Ronen

**Affiliations:** Department of Radiology and Biomedical Imaging, University of California San Francisco, San Francisco, California, United States of America; Texas A&M University, United States of America

## Abstract

Histone deacetylase (HDAC) inhibitors have emerged as effective antineoplastic agents in the clinic. Studies from our lab and others have reported that magnetic resonance spectroscopy (MRS)-detectable phosphocholine (PC) is elevated following SAHA treatment, providing a potential noninvasive biomarker of response. Typically, elevated PC is associated with cancer while a decrease in PC accompanies response to antineoplastic treatment. The goal of this study was therefore to elucidate the underlying biochemical mechanism by which HDAC inhibition leads to elevated PC. We investigated the effect of SAHA on MCF-7 breast cancer cells using ^13^C MRS to monitor [1,2-^13^C] choline uptake and phosphorylation to PC. We found that PC synthesis was significantly higher in treated cells, representing 154±19% of control. This was within standard deviation of the increase in total PC levels detected by ^31^P MRS (129±7% of control). Furthermore, cellular choline kinase activity was elevated (177±31%), while cytidylyltransferase activity was unchanged. Expression of the intermediate-affinity choline transporter SLC44A1 and choline kinase α increased (144% and 161%, respectively) relative to control, as determined by mRNA microarray analysis with protein-level confirmation by Western blotting. Taken together, our findings indicate that the increase in PC levels following SAHA treatment results from its elevated synthesis. Additionally, the concentration of glycerophosphocholine (GPC) increased significantly with treatment to 210±45%. This is likely due to the upregulated expression of several phospholipase A2 (PLA_2_) isoforms, resulting in increased PLA_2_ activity (162±18%) in SAHA-treated cells. Importantly, the levels of total choline (tCho)-containing metabolites, comprised of choline, PC and GPC, are readily detectable clinically using ^1^H MRS. Our findings thus provide an important step in validating clinically translatable non-invasive imaging methods for follow-up diagnostics of HDAC inhibitor treatment.

## Introduction

The histone deacetylase (HDAC) enzymes catalyze the removal of acetyl groups from the epsilon-amine of lysine residues of histone tails. The opposing actions of HDACs and histone acetyltransferases (HATs) dictate the acetylation status of histone lysine residues, regulating gene transcription through chromatin packaging [1], [2]. HDAC enzymes also modify at least 50 non-histone proteins of known biological function, including transcription factors, transcription regulators, signal transduction mediators, chaperone proteins and inflammation mediators [2], resulting in altered stability and activity of the target proteins. Interestingly, epigenetic modulation of genes involved in tumor initiation and progression has been linked to altered expression or mutation of genes encoding HAT, HDAC or their binding partners [3], [4]. Additionally, aberrant recruitment of HDAC enzymes has been associated with the development of certain human cancers. Consistent with these findings, HDAC inhibitors (HDACi), a recently developed class of anticancer drugs, have been shown to induce differentiation, growth arrest and apoptosis in treated cells and tumors, while normal cells are relatively resistant to HDACi effects [2], [5]. Vorinostat (suberoylanilide hydroxamic acid; SAHA) has been approved by the FDA for treatment of cutaneous T-cell lymphoma, while newly developed HDAC inhibitors are having success in clinical trials in various tumor types, including breast cancer, acute myeloid leukemia, and Hodgkin lymphoma [3], [6]–[8].

Clinical use of therapeutic drugs is significantly enhanced when a non-invasive means of longitudinally monitoring treatment efficacy is available. Traditional imaging methods may not be adequate for rapid monitoring of molecular drug action and response, since in many cases molecular drug action results in tumor stasis rather than shrinkage [9], [10]. Magnetic resonance spectroscopy (MRS) presents a non-invasive and non-destructive method that can provide biochemical information of normal and tumor tissues, presenting pharmacodynamic biomarkers of oncogenesis and response to anticancer therapies [11]–[14]. As emerging targeted therapies continue to be used more prevalently, the development of accompanying biomarkers is equally important. For this reason, identifying novel biomarkers of target inhibition that are detectable by non-invasive methods is essential for determining the efficacy of treatment and correlation with antitumor effects. Furthermore, the validation of a biomarker through the characterization of the underlying biochemical mechanism(s) provides context necessary for effective interpretation, and may suggest the strengths and limitations of the biomarker.

We have previously used MRS to develop and mechanistically validate biomarkers of response to emerging targeted therapies [15]–[21]. In particular, we have shown that treatment with the HDAC inhibitor SAHA is associated with increased PC levels [22]. Consistent with this finding, administration of LAQ-824 or Belinostat, two other HDAC inhibitory compounds, led to increased PC levels not only in cells but also in tumors *in vivo* [23], [24]. These observations are particularly interesting, as they run counter to conventional wisdom. Typically, an increase in the endogenous choline-containing metabolites is associated with cell transformation, while a decrease is observed in response to cytotoxic treatments [11]–[14], [25]–[27]. Importantly though, treatment with 17AAG, an inhibitor of the heat shock protein 90 (HSP90), also leads to increased PC levels [16], [28], and HSP90 is one of the proteins affected by HDAC inhibition [22]. We have recently shown that in the case of MCF7 cells, the increase in PC following 17AAG treatment is due to an increase in expression of the choline transporter SLC44A1 [29]. Accordingly, HSP90 inhibition and the resulting choline transport upregulation offers a possible mechanism by which PC levels increase with SAHA. The goal of this study was therefore to assess the mechanisms by which SAHA elevates PC levels in MCF7 breast cancer cells. To this end, ^1^H, ^31^P and ^13^C MRS of MCF7 cells labeled with [1,2-^13^C]choline was performed to characterize choline metabolism and its modulation following treatment with the HDAC inhibitor SAHA. In parallel, the expression and activity of enzymes involved in choline metabolism were also probed. We found that, similar to HSP90 inhibition, SAHA treatment led to an increase in the expression of the choline transporter SLC44A1, but that in addition the expression and activity of choline kinase α`s also increased in treated cells.

## Materials and Methods

### Cell Culture

MCF7 cells were obtained from ATCC. Unique DNA “fingerprint” identities (i.e., variable number tandem repeat PCR products) have been established for the cell line used in this study, and the identity of the cell line was confirmed in association with its use in the experiments described here.

Cells were cultured in DMEM supplemented with 10% heat-inactivated fetal bovine serum, 2 mM L-glutamine, 100 units/mL penicillin and 100 µg/mL streptomycin. For MRS labeling studies, custom DMEM without choline (UCSF Cell Culture Facility) was supplemented with [1,2-^13^C]choline chloride at a final concentration of 28 µM (the concentration normally present in DMEM). For all experiments, cells were harvested in their logarithmic phase of proliferation.

For HDAC inhibition, cell cultures were incubated with 10 µM SAHA (courtesy of Drs. W. Bornmann and A. Pal, University of Texas M.D. Anderson Cancer Center for initial studies, and Cyman Chemical Company for subsequent studies; findings were within experimental error), replenishing drug and medium every 24 h. The final concentration of DMSO was 1∶1000 in culture medium.

### Cell Proliferation

The effect of drug treatment on cell proliferation was determined using the WST-1 reagent assay (Roche). Cells were seeded in 96-well plates and treated for 4 to 48 h with 0, 5, 10, and 20 µM SAHA. After treatment, WST-1 reagent was incubated in wells for 1 h and cell viability was determined by quantification of absorbance at 440 nm using a spectrophotometer (Tecan).

### HDAC Inhibition

The effect of SAHA on HDAC activity was determined using the Fluor de Lys fluorometric assay (Biomol), following the manufacturer’s instructions. Cells were seeded in 96-well plates and incubated with SAHA for 48 h. The Fluor de Lys substrate was added for 1.5 h, medium was transferred to adjacent wells, and cells were rinsed with PBS. Fluor de Lys developer was added to both the wells containing cells and medium. After 20 min, fluorescence was measured at 460 nm using a spectrofluorometer (Tecan).

### Cell Cycle and Cell Size Analysis

Control and treated cells were harvested by trypsinization, washed in PBS and fixed in 95% ethanol. After fixation cells were washed and resuspended in PBS supplemented with 10 µg/ml RNase A (Sigma) and 20 µg/mL propidium iodide (Biotium). After 30 minutes of staining, cells were analyzed using a fluorescence-activated cell sorting (FACS) Calibur flow cytometer and CellQuest Pro software (BD Biosciences). Single cells were gated away from clumped cells by using forward light scattering on an FL2-width versus FL2-area dot plot. Percentages of cells in the G1, S, and G2/M phases were determined by plotting a histogram of FL2-A. Cell size distribution was determined by Coulter counter. Approximately 5×10^6^ cells per sample were diluted into 10 mL of Isoton solution in a clean cuvette. Coulter counter readings provided cell number and cell size.

### MRS Acquisition and Analysis

For initial MRS studies, MCF7 cells were treated for 48 h with 0, 5, 10 and 20 µM SAHA (n = 2). For mechanistic studies, MCF7 cells were treated for 48 h with 10 µM SAHA. Culture medium was replaced with [1,2-^13^C]choline-labeled medium 6 h prior to extraction. Cells (∼3×10^7^) were extracted using the dual-phase extraction method, as previously described [22], [30]. Briefly, cells were harvested and washed with saline to remove residual medium. Cells were fixed in 10 mL ice-cold methanol, 10 mL ice-cold chloroform was added, followed by 10 mL ice-cold deionized water. Following phase separation and solvent removal by lyophilization, the aqueous fraction was reconstituted in 400 µL deuterium oxide (D_2_O) for ^1^H and ^13^C measurements. Before performing ^31^P measurements, EDTA was added to a final concentration of 10 µM. MR studies were performed on a 600-MHz Varian spectrometer at 25°C. ^13^C and ^31^P spectra were obtained using a 30° acquisition pulse with proton decoupling and a 3-s relaxation delay. ^1^H spectra were acquired with a 90° pulse and 3-second relaxation with suppression of the water signal. Metabolite concentrations were determined by integration, normalized to the area of the external reference (TMS, ^1^H and ^13^C; MDPA, ^31^P) and cell number, and corrected for saturation effects.

### Choline Kinase Activity

Experiments were performed to assay the total cellular activity of choline kinase as previously described [31], [32]. In brief, cell pellets (2×10^7^) were resuspended in 540 µL lysis buffer containing 100 mM Tris-HCl (pH 8.0), 10 mM DTT and 1 mM EDTA in D_2_O. Lysate was homogenized on ice by repeated passage through fine-tipped needle (27G 1/2) and further disrupted by sonication 10×1s at 20 kHz. The homogenate was centrifuged at 16,000 rpm for 30 minutes at 4°C. The choline kinase activity of the supernatant fraction was measured immediately after addition of 60 µL reaction mixture (final concentrations: 50 mM Tris-HCl (pH 8.0), 5 mM choline chloride, 10 mM ATP and 10 mM MgCl_2_). MR experiments were performed at 25°C on a 600-MHz Varian spectrometer equipped with a 5-mm broadband observe probe. ^1^H MR spectra were obtained over the course of 1 hour using 90° acquisition pulses and 3-second relaxation delay with water presaturation. Choline and PC concentrations were simultaneously measured from peak areas. Choline kinase activity was determined by regression analysis of the linear portion of the time course of PC formation.

### Cytidylyltransferase Activity

Experiments were performed to measure the activity of phosphatidylcholine-specific CTP:phosphocholine cytidylyltransferase, as previously described [29]. In brief, cell pellets (2×10^7^) were resuspended in 540 µL lysis buffer containing 50 mM HEPES (pH 7.0), 5 mM EDTA, 5 mM EGTA, 1 µL/mL protease inhibitor cocktail (Calbiochem) and 5.5 mM sodium bisulfite. Lysate was repeatedly passed through a fine-tipped needle (27G 1/2) for homogenization and was sonicated 10×1s at 20 kHz for membrane solubilization. The homogenate was centrifuged at 16,000 rpm for 30 minutes at 4°C. The cytidylyltransferase activity of the supernatant fraction was measured immediately after addition of 60 µL reaction mixture (final concentrations: 50 mM Tris-HCl (pH 8.0), 10 mM cytidine triphosphate (CTP), 5 mM PC, 5 mM DTT and 25 mM MgCl_2_). MR experiments were performed at 33°C on a 600-MHz Varian spectrometer equipped with a 5-mm broadband observe probe. ^31^P MR spectra were obtained using 30° acquisition pulse with proton decoupling, 2.6-s relaxation delay and 128 transients per FID. PC and CDP-choline concentrations were simultaneously measured from peak areas normalized to the area of the external reference (MDPA) and cell number, and corrected for saturation effects. Cytidylyltransferase activity was determined by regression analysis of the linear portion of the time course of CDP-choline formation.

### Phospholipase A Activity

The activities of phospholipase A_1_ and A_2_ were determined using the EnzChek phospholipase A assay kits (Invitrogen) according to manufacturer’s instructions. The fluorescent cleavage product was measured (495 nm excitation, 515 nm emission) by an Infinite M200 fluorescence spectrophotometer (Tecan).

### Immunoblotting

Control and treated cells were lysed using Cell Lysis Buffer (Cell Signaling). Lysates, were run on 4%–20% Tris-HCl Gels (Bio-Rad) by SDS-PAGE and electrotransferred onto PVDF membranes (Millipore). Blots were blocked and incubated with primary antibodies, anti-PLA2G4C (Abcam), anti-PLA2G10 (Abnova), anti-ChoK α (Sigma), anti-SLC44A1 (Abcam) or anti-β-Actin (Cell Signaling), then incubated with secondary antibody anti-IgG HRP-linked antibody (Cell Signaling). Immunocomplexes were visualized using ECL Western Blotting Substrate (Pierce). Quantification was performed using ImageJ (NIH) by measuring band intensities in scanned blots, and normalizing to loading control. Results are expressed as percentage of controls.

### Statistical Analysis of Immunoblotting, Extract and Activity Assay Results

All results are expressed as mean ± SD and represent an average of three repeats unless otherwise stated. Two-tailed unpaired Student’s *t* test was used to verify statistical significance of the results, with *P*≤0.05 considered to be significant.

### Gene Expression Analysis

Total cellular RNA was isolated from approximately 1×10^7^ cells after 48 h of treatment with SAHA or vehicle (DMSO), using the RNeasy Mini Kit (Qiagen) according to manufacturer’s instructions. RNA quality was determined by Bioanalyzer (Agilent), considering RNA integrity number (RIN) values of ≥8.0 acceptable (most values were 9.5 or higher). Microarray hybridization was performed at the UCSF Genomics Core Laboratories using the Human Gene 1.0 ST assay (Affymetrix). Arrays were analyzed by fluorescence detection using the Agilent GeneArray Scanner (Agilent). Data acquisition was performed using the MicroArray Suite 5.0 software (Affymetrix). Microarray experiments were performed with 4 repeats of each condition. Microarray data is available through the ArrayExpress public repository [EMBL:E-MTAB-339] in compliance with standards of the Microarray Data Gene Expression Society.

## Results

### SAHA Inhibits Cell Proliferation and Elevates Levels of PC and GPC

**Figure 1:**
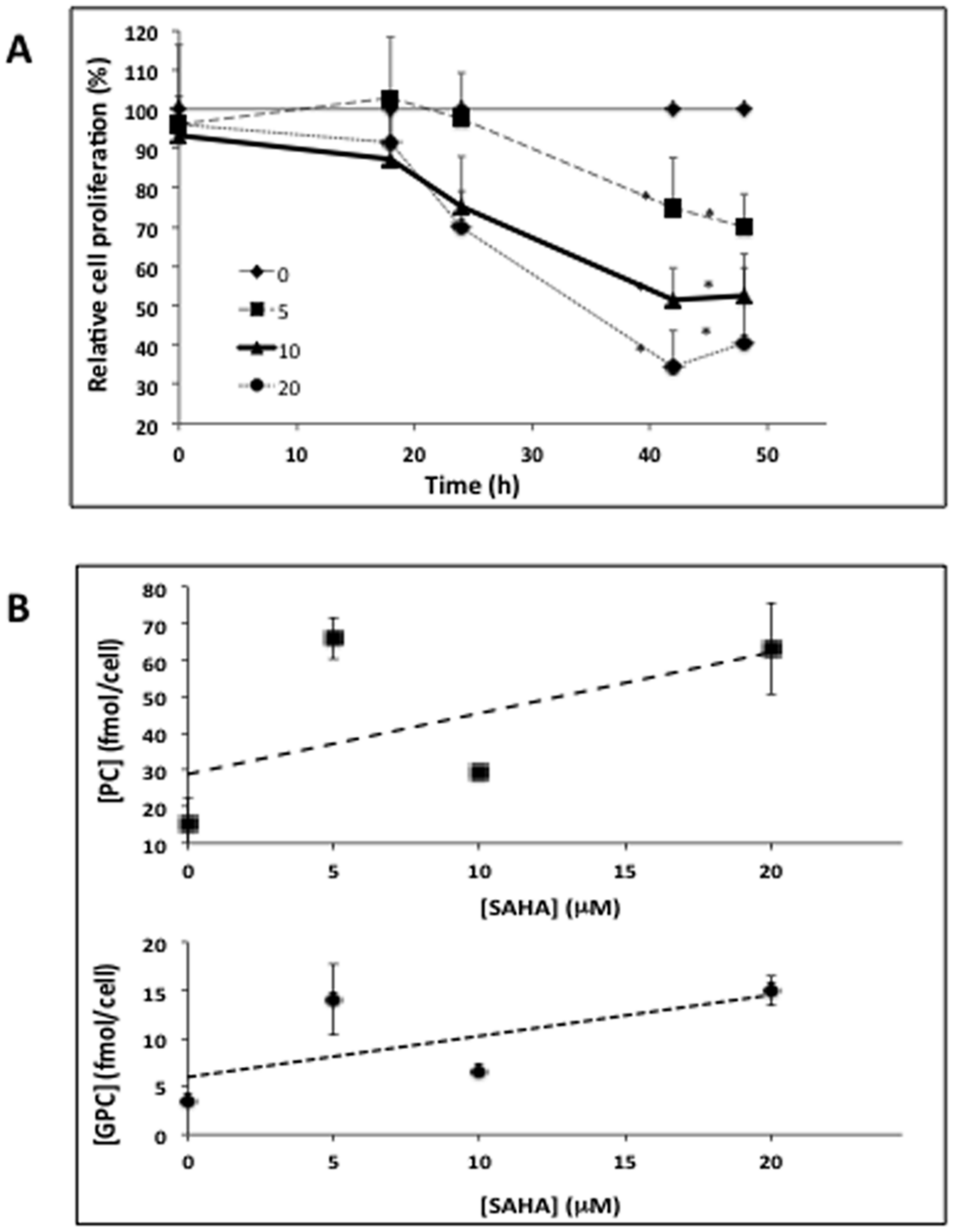
Efficacy of SAHA treatment on cellular proliferation and PC levels. (A) WST-1 assay showing anti-proliferative effects of 0, 5, 10 and 20 µM over a 48-hour treatment period; (B) Cellular PC and GPC levels following treatment with 0, 5, 10 and 20 µM SAHA at 48-hour time point.

**Figure 2:**
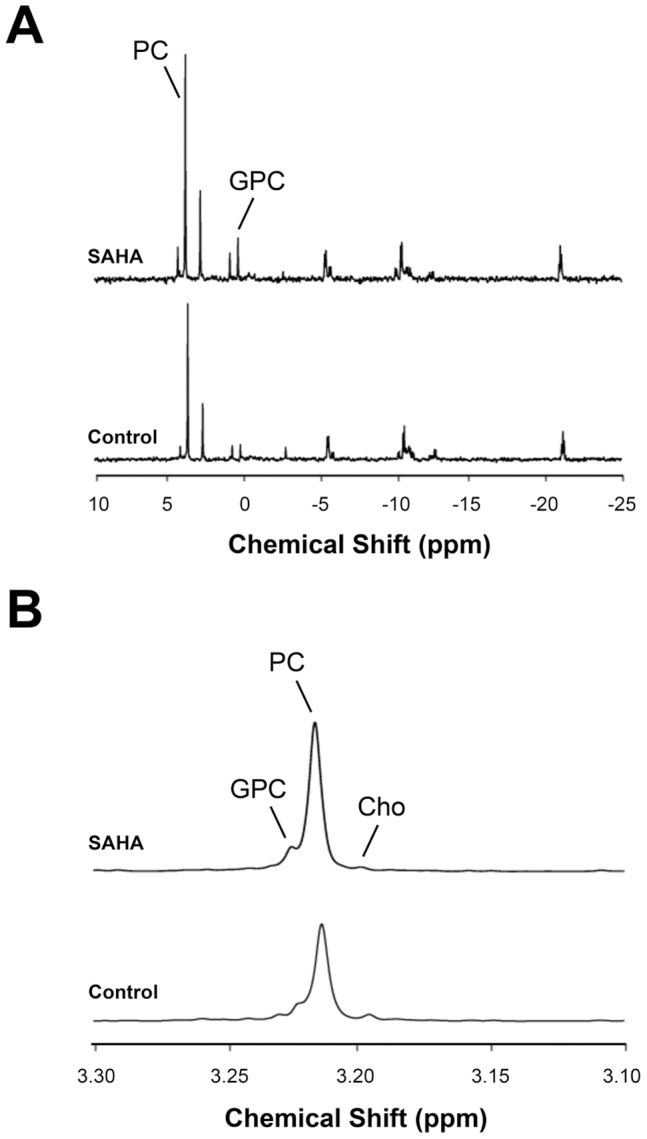
Detection of endogenous metabolites by magnetic resonance spectroscopy. (A) Representative ^31^P spectra of control (bottom) and SAHA-treated (top) MCF7 cell extracts, showing increases in PC and GPC after 48-hour treatment; (B) Representative ^1^H spectra (3.10–3.30 ppm) of choline-containing metabolites, highlighting increased tCho levels after 48-hour treatment.

Figure 1A illustrates the effects of 5, 10 and 20 µM SAHA on MCF7 cells, and demonstrates that all treatment doses resulted in significant inhibition of cell proliferation by 48 h of treatment. Next, we carried out a small scale MRS study to verify previous observations by our lab, which had found increased PC and glycerophosphocholine (GPC) levels in response to SAHA treatment [22]. Our findings are illustrated in Figure 1B, with typical MR spectra displayed in Figure 2. In line with previous observations, cellular PC and GPC levels increased following 48 h of treatment at all SAHA concentrations investigated.

In order to determined the underlying mechanism of this effect we chose to focus further studies on treatment with 10 µM SAHA for 48 h, a dose that led to a reduction in cell proliferation to 50±5% (P<0.001, n = 3) of control (Fig. 1A). The direct deacetylase assay indicated that this treatment reduced HDAC activity from 74±2 pmol deacetylated substrate/hr to 39±4 pmol/hr, or 53±4% of control (P = 0.0002, n = 3). Cell cycle analysis indicated that the fraction of cells in the G1 phase remained unchanged following SAHA treatment (73±2% in control cells and 73±1% in SAHA-treated cells). The fraction of cells in G2 increased following treatment from 8±1% to 17±1% (P<0.002, n = 3), while the fraction of cells in the S phase dropped from 18±2% to 7±1% (P = 0.001). This slight change in cell cycle distribution did not lead to a significant change in average cell size following SAHA treatment (control: 19.2±3.8 µm, SAHA-treated: 18.9±3.2 µm).

^31^P MRS demonstrated that PC increased to 129±7% of control (P = 0.006, n = 3; Fig. 2A). Control cells had 17.8±1.2 fmol PC/cell, while SAHA-treated cells had 22.5±1.9 fmol PC/cell. Additionally, the concentration of GPC increased significantly with SAHA treatment to 210±45% of control, from 1.6±0.3 fmol GPC/cell to 3.7±0.4 fmol GPC/cell (P = 0.01, n = 3). The increased levels of choline-containing metabolites were also evident in ^1^H MR spectra (Fig. 2B). Total choline (tCho), a peak that consists of choline (Cho), PC and GPC significantly increased from 19.7±1.5 fmol/cell to 27.2±1.1 fmol/cell with treatment, or 138±9% of control (P = 0.002, n = 3), consistent with the ^31^P MRS findings.

### SAHA Increases Choline Uptake

**Figure 3:**
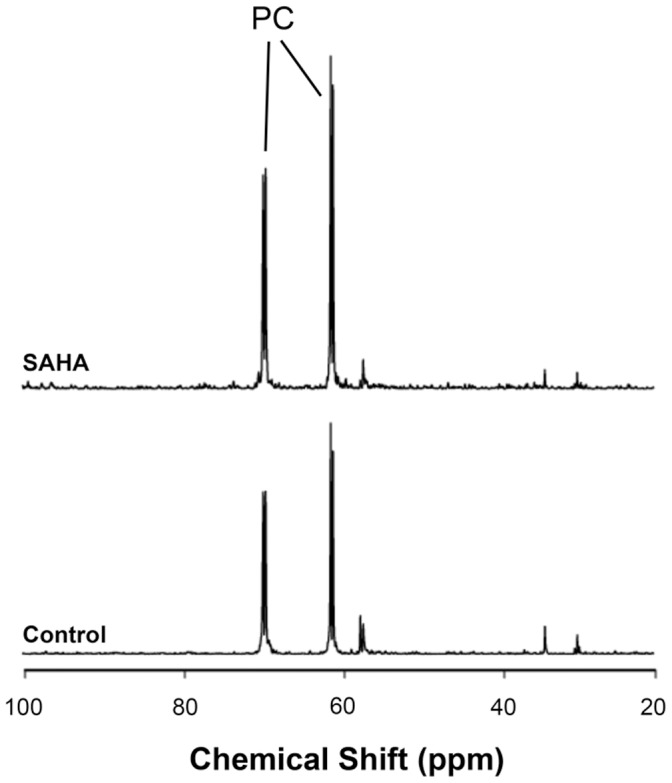
Measurement of choline uptake by magnetic resonance spectroscopy. Representative ^13^C spectra of control (bottom) and SAHA-treated (top) MCF7 cell extracts labeled with [1,2-^13^C]choline, showing increased uptake and incorporation of labeled choline as [1,2-^13^C]PC.

To evaluate the contribution of *de novo* synthesis to increased endogenous PC levels, the uptake of [1,2-^13^C]choline was measured after short-term incubation (Fig. 3). ^13^C MR spectra show PC peaks from the incorporation and phosphorylation of labeled choline. The level of ^13^C-labeled PC was significantly higher in treated cells representing 154±19% of control. The labeled PC pool in control cells was 8.5±1.5 fmol/cell, and 13.1±0.9 fmol/cell in treated cells (P = 0.01, n = 3). It is worth noting that this increase in *de novo* PC was within standard deviation of the increase in total endogenous PC levels. Relative to the total PC levels determined from the ^31^P spectra, 47±6% of the PC pool was ^13^C-labeled in control cells and 59±8% of the pool was labeled in SAHA-treated after 6 hours of labeling, with no significant difference in the percentage of labeling between control and treated cells (P = 0.13, n = 3). Over this time period, no label was distributed beyond PC into PtdCho, GPC or other phospholipids, which is consistent with previous studies [29].

Interestingly, there is no [1,2-^13^C]choline peak visible in the ^13^C spectrum. The concentration of labeled choline does not reach the detection limit, which suggests that choline is phosphorylated shortly after being transported into the cell. The relative rates of the two steps involved in PC synthesis can be estimated using the signal-to-noise ratio of the PC peaks (SNR values ≥20) [33]. Thus, choline phosphorylation is at least 20-fold faster than choline transport, indicating that transport is the rate-limiting step in PC synthesis in MCF7 cells.

### SAHA Modulates Activities of Enzymes Involved in Choline Metabolism

**Figure 4:**
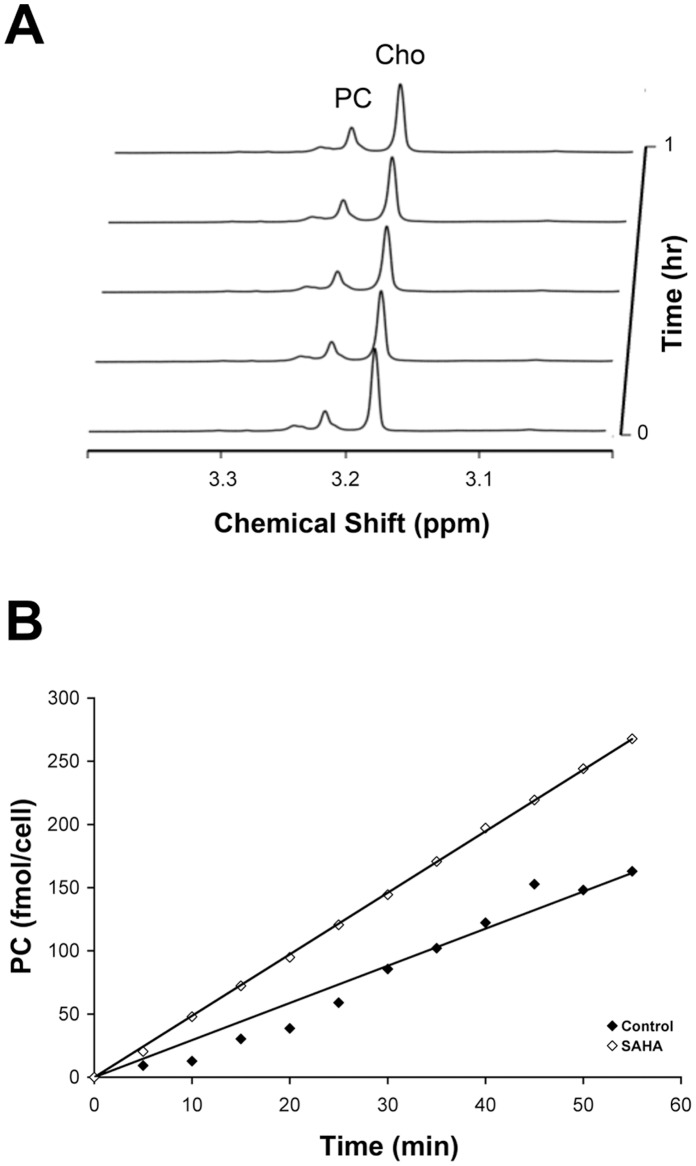
Quantification of choline kinase activities. (A) Simultaneous detection of PC and choline peaks in MCF7 cell cytosolic preparations over 1 hour; (B) Time courses of PC production in control and SAHA-treated cells in representative experiments, showing increased choline kinase activity with treatment.

In order to explain the observed changes in choline-containing metabolite concentrations, we analyzed the activities of choline kinase and CTP: phosphocholine cytidylyltransferase, the enzymes directly involved in PC production and consumption respectively. The cellular activity of choline kinase was measured by ^1^H NMR assay in cell lysates. Figure 4 shows a representative time course of the choline kinase-mediated PC production and choline consumption. These assays revealed a choline kinase rate of 3.6±0.8 fmol PC/cell per hr in control cells and an increase to 6.3±1.4 fmol PC/cell per hr in treated cells (177±31% of control; P = 0.04).

**Figure 5:**
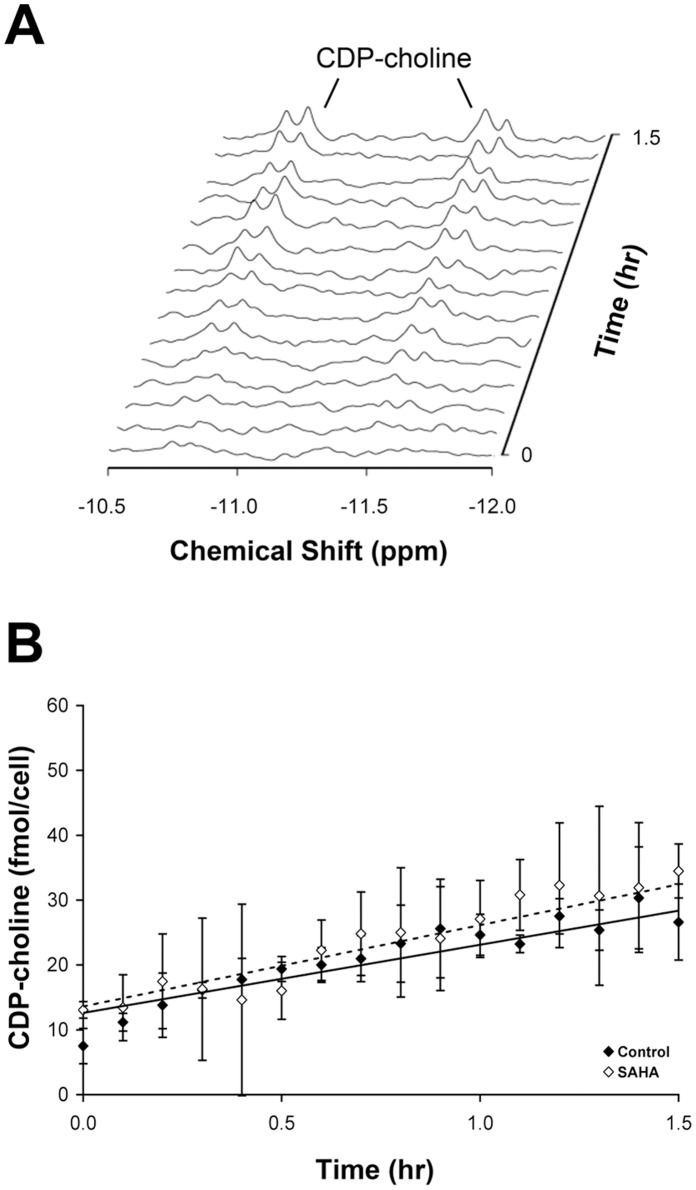
Quantification of CTP:phosphocholine cytidylyltransferase activities. (A) Buildup of CDP-choline peaks in MCF7 cell lysates over 1.5 hours; (B) Time courses of CDP-choline production in control and SAHA-treated cells, showing no significant change in cytidylyltransferase activity with treatment.

The activity of CTP:phosphocholine cytidylyltransferase was measured in total cell lysates by ^31^P NMR assay. These assays revealed a rate of 10.0±2.2 fmol CDP-choline/cell per hr and no significant change in treated cells (P = 0.15). Figure 5 shows a representative time course of the cytidylyltransferase-mediated CDP-choline production.

Fluorimetric assessment of PLA activities measured in total cell lysates revealed a 162±18% (P = 0.03) increase in PLA_2_, while no significant change was seen in PLA_1_ (115±12%; P = 0.13).

### SAHA Alters Expression of Genes Associated with Metabolic Modulation

To verify that the increases in enzyme activities are due to elevated levels of enzymes, we determined the relative expressions of annotated genes associated with choline metabolism [34] by mRNA microarray analysis (Table 1). SAHA treatment resulted in changes in gene expression of several proteins directly involved in choline metabolic pathway. Expression of intermediate-affinity choline transporters SLC44A1 and SLC44A3 increased (144% and 126%, respectively), while SLC44A2 decreased (69%). No significant changes were observed in high-affinity choline transporter SLC5A7 or low-affinity polyspecific organic-cation transporters SLC22A1-3. Expression of choline kinase α (CHKA) increased (161%), while choline kinase β (CHKB) decreased (77%). No significant changes were seen in the expression of CTP:phosphocholine cytidylyltransferase 1α and β (PCYT1A and PCYT1B). Expression of several PLA_2_ isoforms (PLA2) increased significantly, most notably PLA2G4C (271%) and PLA2G10 (366%). There were conflicting changes in expression of GDPDs, with an increase in GDPD1 and a decrease in GDPD3. It is worth noting that the gene sequences of phosphatidylcholine-specific phosholipase C has not yet been determined, and therefore was not included in the microarray analysis.

**Table 1:** Summary of microarray data for relative changes of expression for enzymes involved in choline metabolism.

Function	Gene Symbol	Gene Title	MA	% Control	FDR
Choline Transporters	SLC5A7	solute carrier family 5, member 7	−0.13	91	0.3
	**SLC44A1**	**solute carrier family 44, member 1**	**0.53**	**144**	**1.10E–05**
	**SLC44A2**	**solute carrier family 44, member 2**	**−0.54**	**69**	**9.70E–06**
	**SLC44A3**	**solute carrier family 44, member 3**	**0.33**	**126**	**0.014**
	SLC44A4	solute carrier family 44, member 4	0.04	103	0.75
	SLC44A5	solute carrier family 44, member 5	0.08	106	0.54
	SLC22A1	solute carrier family 22, member 1	0.07	105	0.66
	SLC22A2	solute carrier family 22, member 2	0.00	100	0.99
	SLC22A3	solute carrier family 22, member 3	−0.01	99	0.96
Choline Kinases	**CHKA**	**choline kinase alpha**	**0.69**	**161**	**3.00E–06**
	**CHKB**	**choline kinase beta**	**−0.38**	**77**	**0.0075**
Cytidylyltransferases	PCYT1A	phosphocholine	−0.01	99	0.96
		cytidylyltransferase 1 alpha			
	PCYT1B	phosphcholine	0.08	106	0.53
		cytidylyltransferase 1 beta			
Choline Phosphotransferase	**CHPT1**	**choline phosphotransferase 1**	−**0.52**	**70**	**0.00036**
Phospholipase A	PLA1A	phospholipase A1 member A	0.11	108	0.48
	PLA2G1B	phospholipase A2, group IB	0.24	118	0.074
	PLA2G2A	phospholipase A2, group IIA	−0.16	90	0.13
	PLA2G2C	phospholipase A2, group IIC	0.05	104	0.8
	PLA2G2D	phospholipase A2, group IID	0.03	102	0.83
	PLA2G2E	phospholipase A2, group IIE	−0.22	86	0.22
	PLA2G3	phospholipase A2, group III	−0.06	96	0.74
	**PLA2G4C**	**phospholipase A2, group IVC**	**1.44**	**271**	**4.50E–08**
	PLA2G4E	phospholipase A2, group IVE	−0.04	97	0.83
	PLA2G4F	phospholipase A2, group IVF	−0.22	86	0.058
	PLA2G5	phospholipase A2, group V	−0.13	91	0.53
	**PLA2G6**	**phospholipase A2, group VI**	**0.27**	**121**	**0.042**
	PLA2G7	phospholipase A2, group VII	−0.04	97	0.83
	**PLA2G10**	**phospholipase A2, group X**	**1.87**	**366**	**1.20E–06**
	PLA2G12A	phospholipase A2, group XIIA	0.05	104	0.61
	PLA2G12B	phospholipase A2, group XIIB	0.12	109	0.3
	**PLA2G15**	**phospholipase A2, group XV**	**0.74**	**167**	**7.50E–06**
	**PLA2G16**	**phospholipase A2, group XVI**	**0.57**	**148**	**8.90E–05**
Phospholipase D	**PLD1**	**phospholipase D family, member 1**	**1.95**	**386**	**4.50E–09**
	PLD2	phospholipase D family, member 2	0.11	108	0.52
	**PLD3**	**phospholipase D family, member 3**	**1.20**	**230**	**2.20E–08**
	PLD4	phospholipase D family, member 4	0.11	108	0.59
	PLD5	phospholipase D family, member 5	0.26	120	0.054
	**PLD6**	**phospholipase D family, member 6**	−**0.45**	**73**	**1.00E–04**
Lysophospholipase	**LYPLA1**	**lysophospholipase I**	−**0.62**	**65**	**1.50E–05**
	**LYPLAL1**	**lysophospholipase-like 1**	**1.13**	**219**	**4.80E–07**
	LYPLA2	lysophospholipase II	0.20	115	0.061
Glycerophosphocholine	**GDE1**	**glycerophosphodiester**	**0.32**	**125**	**0.017**
Phosphodiesterase		**phosphodiesterase 1**			
	**GDPD1**	**glycerophosphodiester**	**2.07**	**420**	**7.00E–09**
		**phosphodiesterase domain 1**			
	GDPD2	glycerophosphodiester	−0.21	86	0.18
		phosphodiesterase domain 2			
	**GDPD3**	**glycerophosphodiester**	−**1.47**	**36**	**1.70E–07**
		**phosphodiesterase domain 3**			
	GDPD5	glycerophosphodiester	0.03	102	0.89
		phosphodiesterase domain 5			

Genes for which log2<3 were considered as not expressed.

**Figure 6:**
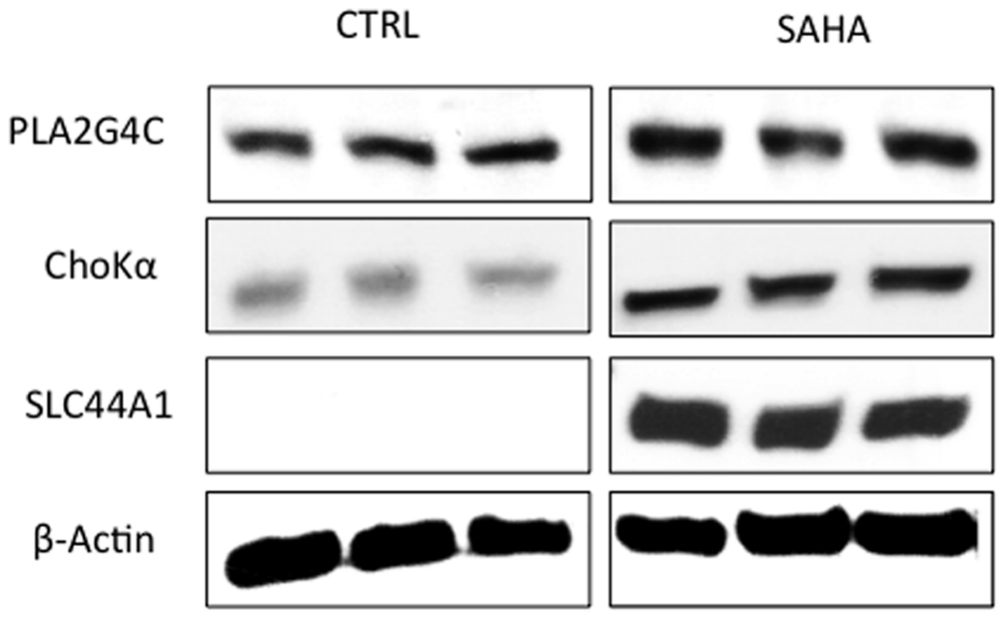
Immunoblots probing the protein expression of PLA2G4C, ChoKα SLC44A1. β-actin served a loading control.

To further confirm the most significant microarray findings, Western blot analysis was used to monitor the protein levels of SLC44A1, ChoK α, PLA2G4C and PLA2G10. Our findings are illustrated in Figure 6. Quantification of the immunoblots indicated that the expression of SLC44A1, CHKA, and PLA2G4C all increased significantly, in line with mRNA microarray analysis. SLC44A1 increased significantly from below detection level in control cells, to clearly detectable levels in treated cells. ChoK α increased to 270±27% of control (P  = 0.01) and PLA2G4C increased to 138±19% of control (P = 0.03). PLA2G10 also increased to 143±23%, but this increase did not reach statistical significance (data not shown).

## Discussion

Novel therapeutic approaches are increasingly developed to target specific molecular genetic events associated with cancer. These advances will lead to more personalized cancer treatment and are expected to result in improved response and reduced toxicity. However, several challenges remain. Most significantly, many targeted therapies result in tumor stasis rather than shrinkage. Consequently, there is a critical need for non-invasive functional imaging biomarkers that confirm drug delivery and molecular drug activity at the tumor site.

Previous studies have reported increased PC levels in response to HDAC inhibition in cell lines and in tumors of several cancer types. We showed that PC levels were inversely correlated with HDAC activity in SAHA-treated PC3 cells [22], [23], and show here that a similar trend is detectable in MCF7 cells. A study in colorectal and prostate cells using belinostat – an HDAC inhibitor with an alternative chemical scaffold – also reported an increase in PC, both in cells and in *in vivo* [24]. Although promising, the use of PC as a validated biomarker requires an understanding of the mechanistic underpinning of metabolic modulation. This is particularly important because the change in PC is counter to that observed in response to chemotherapy or treatment with specific inhibitors of oncogenic signaling pathways. As recently reviewed, PC levels are controlled by multiple factors and are typically higher in proliferating cells when compared to cells in which proliferation is inhibited following treatment [34]. Consequently, an elevation in PC, while providing a potential biomarker of drug action, also requires robust mechanistic validation.

Our studies focused on treatment with 10 µM SAHA, a dose that led to a 50% inhibition in cell proliferation, and thus could be considered as mimicking response to treatment. This dose was consistent with a previous study on 8 breast cancer cell lines, in which the IC_50_ ranged from 0.81 to 18.6 µM [35]. Early clinical trials, in which SAHA was administered intravenously, resulted in similar plasma concentrations, although more recent trials, in which SAHA is used in combination with other therapies, report plasma concentrations around 1µM [36].

Drug-induced metabolic changes are the result of altered enzyme activities. Elevated PC levels in response to HDACi reflect the modulation of enzymes involved in the Kennedy pathway of phospholipid biosynthesis [34]. An increase in the endogenous PC pool could be caused by several changes: elevation of choline uptake and phosphorylation, via choline transporters and choline kinases; decrease in PC-to-PtdCho conversion, via cytidylyltransferase and choline phosphotransferase; and/or increase in PtdCho breakdown, via phospholipases C and D.

Our results suggest that PC increases as the result of increased choline transport and phosphorylation. The use of ^13^C-labeled choline provided a means to characterize the incorporation of choline. After short-term choline incubation in SAHA-treated cells, an increase in [1,2-^13^C]PC was observed, indicating that the elevated levels of endogenous PC are the result of elevated PC synthesis via choline transport and choline phosphorylation. Choline can be transported into the cell by several classes of proteins: high-affinity choline transporters (SLC5A7), intermediate-affinity choline transporter-like proteins (SLC44A1, etc.), and low-affinity polyspecific organic-cation transporters (SLC22A1, etc.) [37]. The microarray-based gene expression analysis found significant increases in the expression of the intermediate-affinity choline transporters SLC44A1 and SLC44A3 to 144% and 126% of control and a decrease in the expression of SLC44A2 to 69%, while no significant changes in expression were seen in other choline transporters. The increase in expression of SLC44A1, the most significantly altered transporter, was also confirmed at the protein level.

Choline phosphorylation is catalyzed by choline kinase. The activity of the enzyme depends on which subunits (ChoK α and ChoK β) form the dimer, as a recent study demonstrated that the activity of ChoK α is much greater than that of the β isoform [38]. Our activity assay measuring total cellular ChoK activity indicated an increase in cellular ChoK activity, consistent with significant increases in both ChoK α protein and mRNA levels.

However, interpretation of the ^13^C MR results and previous studies suggest that elevated PC levels following treatment are more likely a result of increased choline uptake, at least in our system. Previous studies in MCF7 cells have reported that choline transport is the rate-limiting step in PC synthesis [33], [39]. In our studies, choline was not visible in the ^13^C spectra in either control or treated cells, indicating that [1,2-^13^C]choline was at an intracellular concentration below detection. The proportion of labeled [PC] to [Cho] is greater than 20 (the lowest measured SNR of PC peaks), suggesting that upon transport of choline into the cell it is immediately phosphorylated by ChoK at a rate that is at least 20 times faster than transport and therefore choline transport is the rate-limiting step in PC synthesis. Taken together, our findings indicate that the increase in PC is therefore primarily due to an overall increase in transporter expression and activity following HDAC inhibition.

Our findings with regard to ChoK α are in line with studies of belinostat that demonstrated that the increase in PC was associated with an induction of ChoK α expression, not only in cells but also in the *in vivo* setting. However, the authors of that study did not report on choline transport, and thus it is unclear whether our observations with regard to transport are specific to our system or are more generally applicable [24].

Increased expression of the choline transporter SLC44A1 was observed in MCF7 cells following treatment with the HSP90 inhibitor 17AAG, which led to an increase in PC both in cells and *in vivo* [16], [29]. The increase in SLC44A1 observed in our study following HDAC inhibition could therefore be mediated by an HSP90-associated mechanism. However, 17AAG treatment had no reported effect on choline transporters SLC44A2 and SLC44A3 or choline kinase, suggesting that HDAC inhibition causes additional HSP90-independent changes to transcriptional regulation. Furthermore, reported results of the effect of HDAC inhibition in the *in vivo* setting have been variable. An increase in PC was observed both in cells and *in vivo* in response to LAQ-824 and belinostat [23], [24]. In contrast, the increase in PC did not translate to the *in vivo* setting in the case of PC3 cells treated with SAHA [40]. In this context, it is worth noting that the rate of choline transport is dependent on extracellular choline concentration. The culture conditions used in these studies used 28 µM of choline, the concentration typically used in culture medium. This is higher than physiological concentration, which ranges from 5 to 10 µM. Since choline transport is undertaken by several transporter classes with different affinities, the relative contributions of specific transporters to the overall transport rate are likely to vary over a range of concentrations. Further studies are therefore needed to fully understand the factors contributing to modulation of choline metabolism in the *in vivo* setting.

An additional metabolic change was observed in the MR results – increased GPC. The increase in GPC following treatment could have been caused by the increased catabolic breakdown of PtdCho by PLA_2_ (and lysophospholipase) and/or the inhibition of GPC phosphodiesterase. Indeed, measurement of total PLA_2_ activity showed a significant increase, and expression of several PLA_2_ isoforms increased. Only a limited number of studies have looked at the modulation of PLA_2_ isoforms with oncogenesis or response to treatment. We have previously found that PLA2G10 was significantly elevated in MCF7 cells following HSP90 inhibition [29]. A study comparing MDA-MD-231 breast cancer cells observed a significant decrease in PLA2G4A - a PLA2 isoform that was not significantly detectable in our cells [41]. In the case of ovarian cells, PLA2G4A as well as PLA2G4C were lower in cancer cell lines compared to normal [31]. The role of each of the PLA2 isoforms in breast and other cancer types thus remains to be fully elucidated.

Further studies are needed to assess our findings in other cancer types in both cell and tumor models. Nonetheless, this study was the first to systematically assess a range of enzymes that, collectively, lead to the increased PC and GPC levels associated with HDAC inhibition. Additional studies are also needed to fully understand the complex interplay between HDAC and choline metabolism. Comparison of results to other similar studies suggest that HSP90 may possibly be responsible for mediating some of the effects observed in our study, including the elevation in SLC44A1 and PLA_2_ [7], [29]. Alternatively, a previous study into the transcriptional regulation of SLC44A1 identified the binding sites of transcription factors E2F and Sp1 within the promoter sequence [42]. It has also been shown that E2F and Sp1 interact directly with HDAC, causing alterations in their activities [43]. E2F and Sp1 are also affected by cell cycle and MAPK pathway, which have been found to be modulated in response to HDAC inhibition [3], [44], [45]. However, besides these reports studies on the regulation of choline metabolism are surprisingly sparse. Accordingly, a mechanism connecting SAHA molecular action with expression of other enzymes involved in choline metabolism remains unaddressed.

In summary, this study is a first step in developing the necessary mechanistic understanding that can lead to establishing clinically applicable non-invasive imaging methods, which can help inform on target modulation and potential response to HDAC inhibitor treatment.
